# Aorto–Right Atrial Fistula Complicating Transseptal Puncture: Integrating Contemporary Imaging and the Structuralist's Toolbox

**DOI:** 10.1016/j.jaccas.2025.106062

**Published:** 2025-11-20

**Authors:** Francesco Giangiacomi, Eric Eeckhout, Xavier Galloo, Stijn Lochy

**Affiliations:** a Department of Cardiology, Vrije Universiteit Brussel (VUB), Universitair Ziekenhuis Brussel (UZ Brussel), Brussels, Belgium; b Department of Clinical Sciences and Community Health, University of Milan, Milan, Italy

**Keywords:** complication, percutaneous coronary intervention, treatment

## Abstract

**Background:**

Aorto–right atrial (RA) fistula is a rare and complex pathological condition, most often occurring as an iatrogenic complication.

**Case Summary:**

We present a case of percutaneous closure of aorto-RA fistula, which resulted from an inadvertent transseptal puncture during atrial fibrillation ablation. Computed tomography with 3-dimensional reconstructions was used to identify the optimal fluoroscopic projection, clarify the anatomy of the fistula, and select the most appropriate closure device. The noncoronary cusp was isolated using the cusp overlap view to optimize device alignment and deployment. A Jugular venous approach in combination with a femoral arterial access allowed us to create an arteriovenous loop. A 6/4-mm Amplatzer Duct Occluder (Abbott) was successfully implanted.

**Discussion:**

Multimodality imaging has a pivotal role to guide the interventional strategy. Technical skills and experience in structural practice are crucial to achieve a good result.

**Take-Home Message:**

Percutaneous closure represents a feasible and effective treatment of aorto-RA fistula.

## Visual Summary

**Figure:**
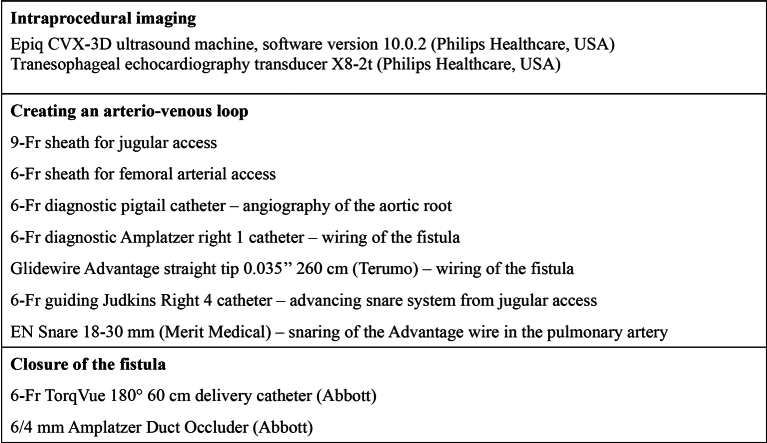

Aorto–right atrial (RA) fistula is a rare and complex pathological condition. It mostly occurs as a complication of cardiac surgery or infectious endocarditis. The clinical presentation can range from an accidental finding in an asymptomatic patient to acute heart failure and cardiogenic shock. Percutaneous closure has been reported to be a feasible and effective therapeutic option, with multimodality imaging playing a crucial role in guiding the interventional strategy and procedure. We present a rare case of an aorto-RA fistula complicating a transseptal puncture performed during atrial fibrillation ablation, successfully treated with percutaneous closure. Key technical and procedural considerations are discussed for optimizing procedural success.

## History of Presentation

A 64-year-old man was admitted to our hospital with bilateral pneumonia and septic shock. On arrival, he appeared pale, tachycardic, and oliguric, with a lactate level of 7.3 mmol/L. He also presented acute-on-chronic kidney disease, with an estimated glomerular filtration rate of 17 mL/min/1.73 m^2^.

## Past Medical History

One year before this admission, he was treated in another center with pulmonary vein radiofrequency isolation for atrial fibrillation; the transseptal puncture had been performed using a BRK needle without transesophageal echocardiographic (TEE) guidance. The procedure was considered successful, and no acute complication was reported.

## Investigations

**Figure 1:**
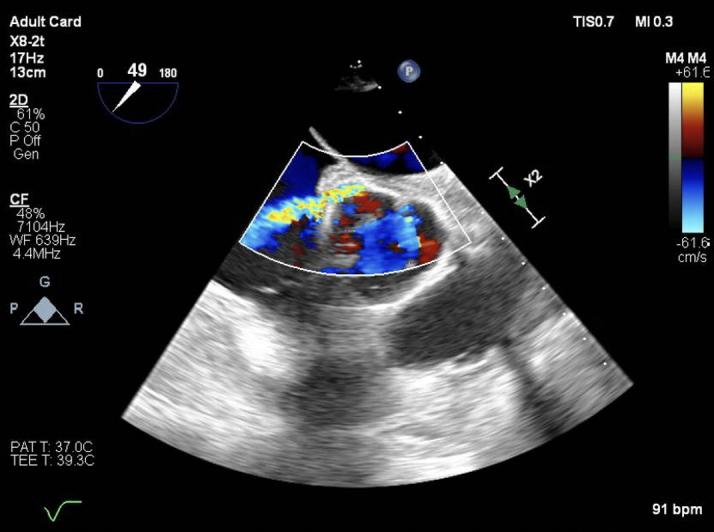
Preprocedural TEE TEE image of a fistula between the noncoronary cusp and the right atrium. TEE = transesophageal echocardiography.

**Figure 2:**
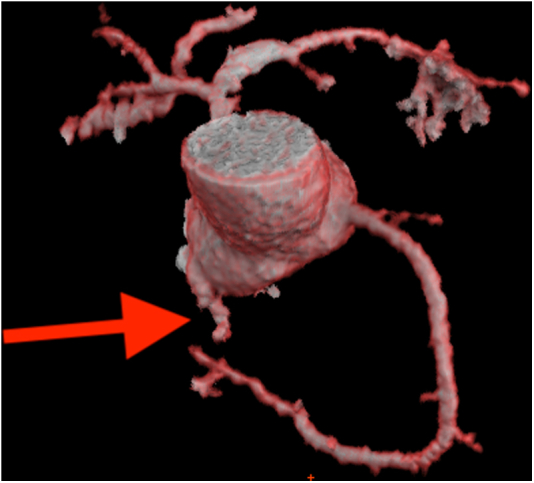
CT 3D Reconstruction CT 3D reconstruction shows a long fistulous tract connecting the noncoronary cusp to the right atrium. The fistula is pointed by the red arrow. CT = computed tomography; 3D = 3-dimensional.

During his stay in the intensive care unit at our hospital, a TEE revealed a fistula between the aortic root—specifically the noncoronary cusp (NCC)—and the RA (Figure 1, Video 1). There were no signs of endocarditis, and the fistula was presumed to be a complication of the transseptal puncture performed during the previous pulmonary vein radiofrequency isolation. To better delineate the anatomy and the shape of the fistula, the patient underwent a computed tomography (CT) scan with 3-dimensional reconstruction (Figure 2). The CT confirmed a long fistulous tract connecting the NCC to the RA.

## Management

After collegial discussion in the heart team, closure of the fistula was indicated because of mild right heart chamber dilation and to prevent the long-term risk of endocarditis. Surgery was deemed high risk because of frailty after a long intensive care unit stay. Given the favorable anatomy demonstrated by multimodality imaging, the heart team decided in favor of percutaneous closure. The intervention was programmed electively after 1 month of cardiac rehabilitation. Blood examinations showed no signs of infection.

**Figure 3:**
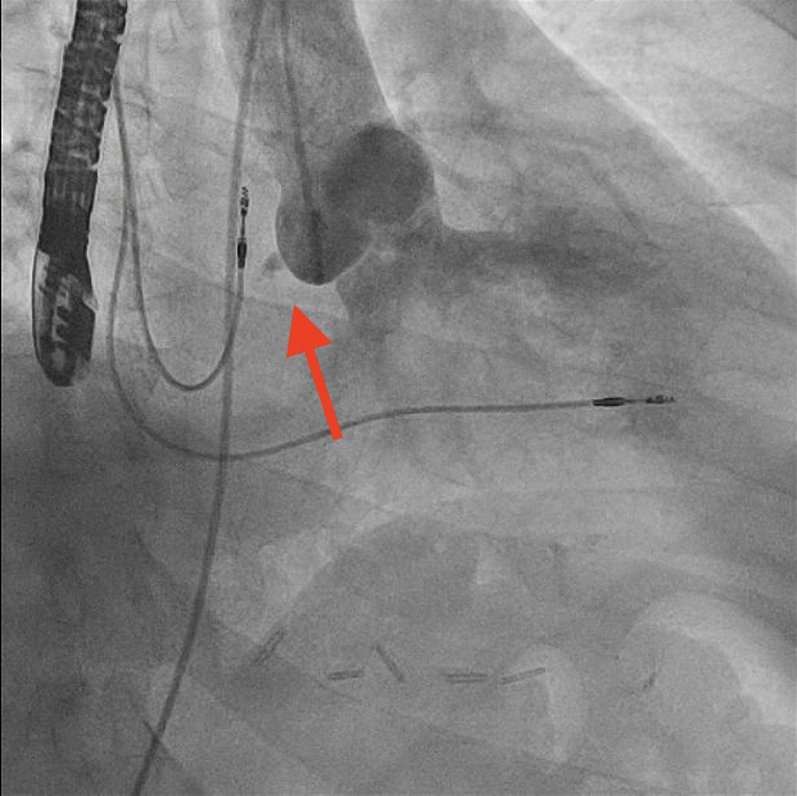
Aortography Aortography in cusp-overlap view, isolating the noncoronary cusp, shows the fistulous tract with the right atrium. A 6F diagnostic pigtail catheter is used to obtain the image. The fistula is pointed by the red arrow.

**Figure 4:**
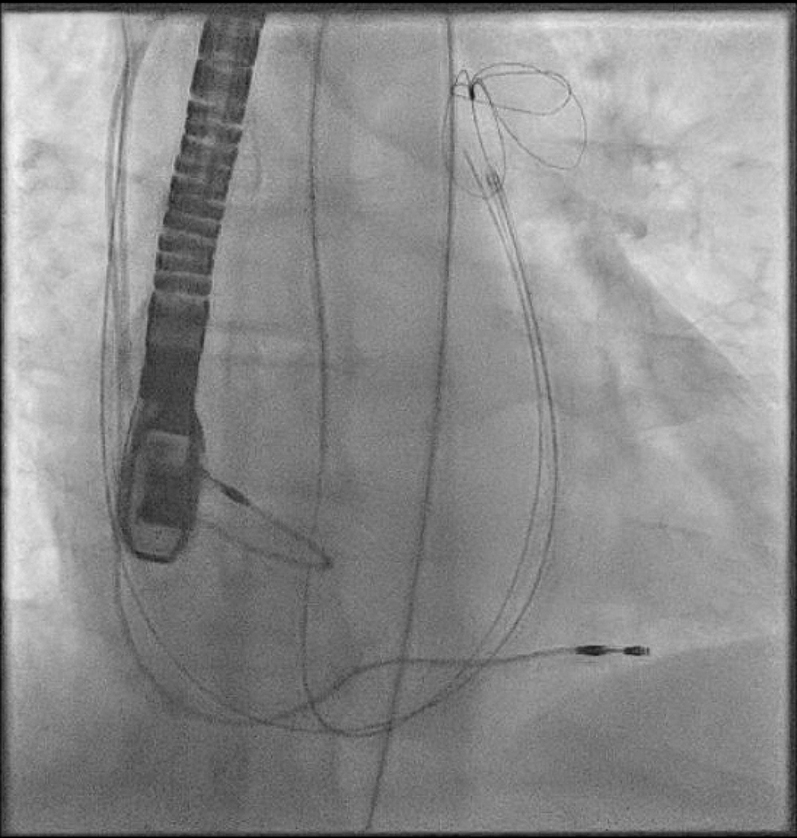
Snaring Snaring of the Advantage wire in the pulmonary artery with an EN Snare 18 to 30 mm (Merit Medical), advanced through the venous access with a 6-F Judkins Right 4 guiding catheter.

**Figure 5:**
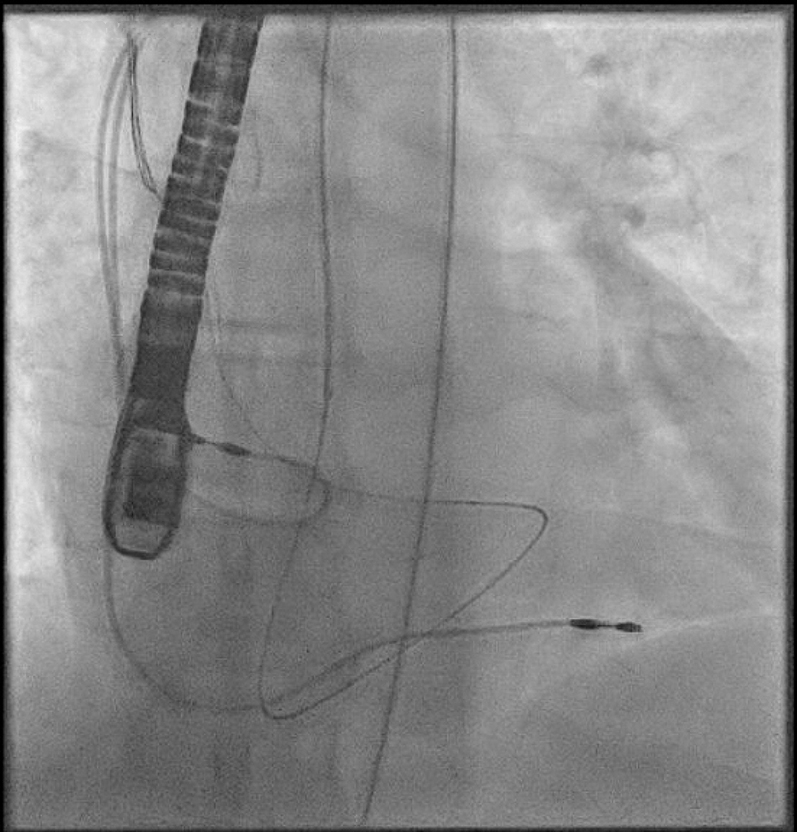
Arteriovenous Loo Externalization of the Advantage wire through the venous access and creation of an arteriovenous loop.

**Figure 6:**
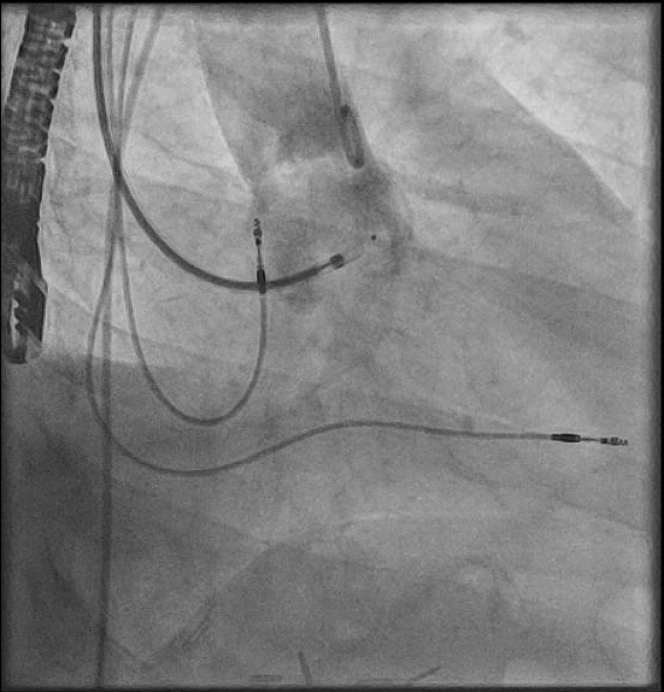
Positioning of the TorqVue Delivery System (Abbott) The TorqVue delivery system is advanced through the venous access, while keeping tension on both the venous and arterial sides of the wire.

**Figure 7:**
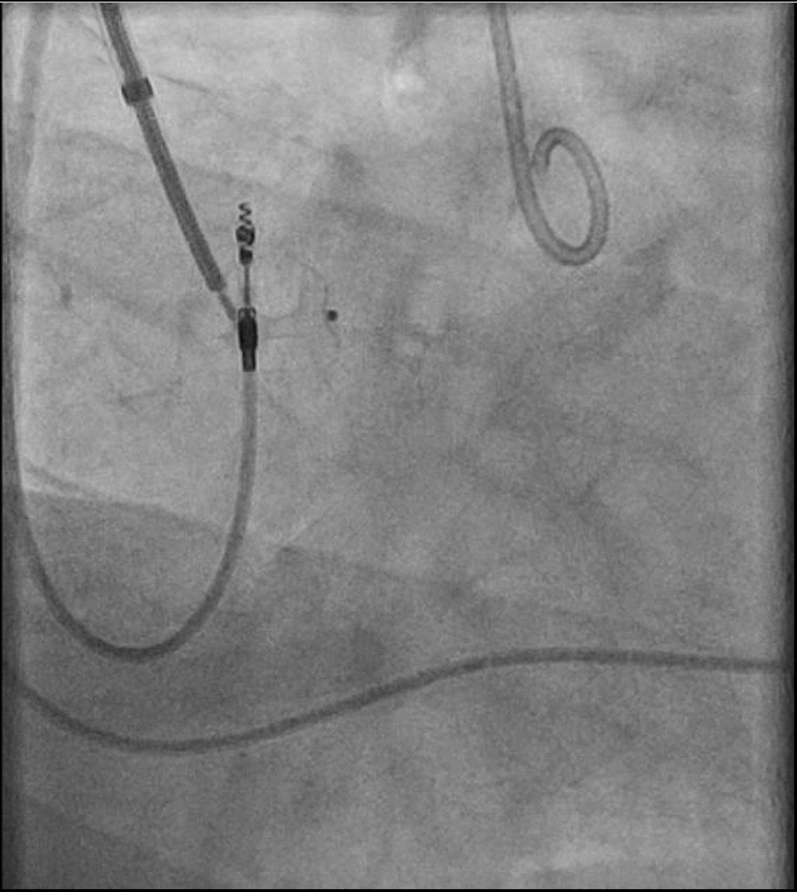
Device Release Release of a 6/4-mm Amplatzer Duct Occluder (Abbott).

**Figure 8:**
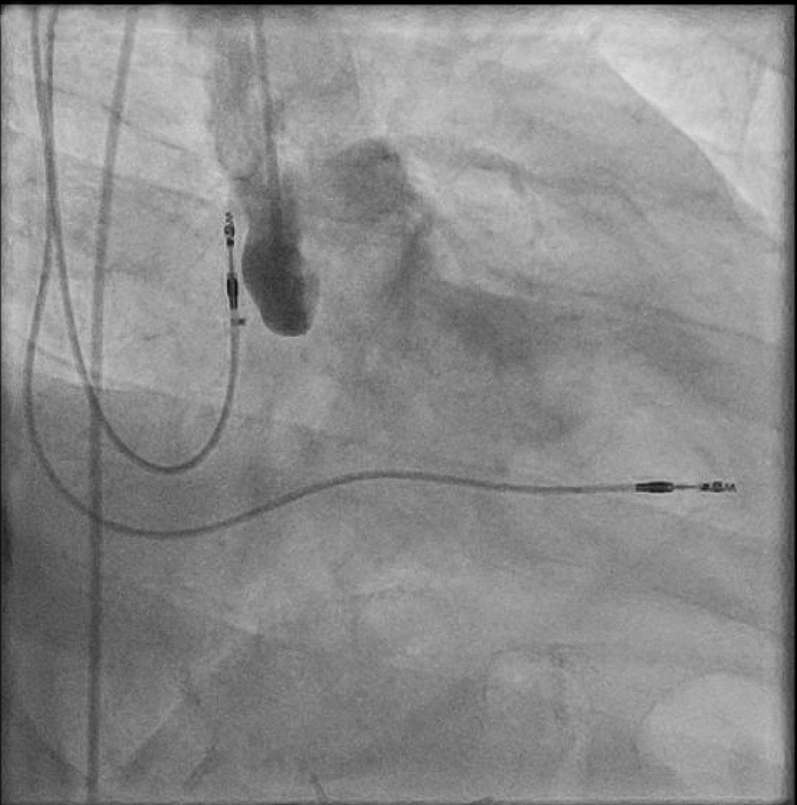
Final Aortography The final aortography shows no residual shunt between the aortic root and the right atrium.

The procedure was performed under general anesthesia. Our setup consisted of a 9-F right internal jugular venous access and a 6-F right femoral arterial access. First, an aortography was performed using a 6-F pigtail diagnostic catheter in the right anterior oblique 28°, caudal 17° view. This projection, mimicking a cusp-overlap view, allowed clear isolation of the NCC and optimal visualization of the fistulous tract (Figure 3). The fistula was easily wired from the aortic root using an Amplatz Right 1 diagnostic catheter and a Glidewire Advantage straight-tip 0.035-inch, 260-cm wire (Terumo) (Video 2). The wire was advanced through the RA and the right ventricle up until the pulmonary artery. Then, a 6-F Judkins Right 4 guiding catheter was advanced through the right jugular venous access, and the wire was snared in the pulmonary artery using an EN Snare 18 to 30 mm (Merit Medical) (Figure 4, Video 3). Snaring is performed in the pulmonary artery, because the reduced space facilitates easier wire capture compared with the RA. Thus, the wire was externalized creating an arteriovenous loop (Figure 5). Over this loop a TorqVue 180° 60-cm delivery catheter 6-F (Abbott) was advanced through the venous access, while keeping tension on both sides of the Advantage wire to optimize the support. Finally, a 6/4-mm Amplatzer Duct Occluder (Abbott) was positioned and released (Figures 6 and 7, Video 4). The intraprocedural TEE showed no interaction between the device and the aortic valve, and the final aortography confirmed the absence of the residual aorto-RA shunt (Figure 8).

## Outcome and Follow-Up

The procedure was performed without complications, and the patient was discharged after 1 week on long-term apixaban because of the previous history of atrial fibrillation; no antiplatelet therapy was added. At the 6-month clinical and echocardiographic follow-up, the patient was stable, with no residual jet between the aortic root and the RA.

## Discussion

Aorto-RA fistula is a rare and complex pathologic condition. It most commonly occurs as a complication of the cardiac surgical procedure, such as valve or aortic surgery, or in the setting of infectious endocarditis. Less frequently, it may result from chest trauma, ruptured sinus of Valsalva aneurysm and aortic dissection, or be of congenital origin.^1^^,^^2^ Aorto-RA fistula has also been described as a rare complication of transseptal puncture during atrial fibrillation ablation, as in our case.^3^^,^^4^ Being a rare condition, data on its incidence and mortality are limited; however, clinical reports have been significantly increasing over the years. Clinical presentation can range from heart failure to cardiogenic shock; less commonly, it may be discovered incidentally in asymptomatic patients.^1^

Percutaneous closure represents an effective therapeutic option and is increasingly adopted for patients at high surgical risk, thanks to technical and procedural advancements. To date, only few cases of percutaneous closure of aorto-RA fistula have been reported, using a variety of techniques and devices. These include Amplatzer septal occluders, Amplatzer vascular plugs, different types of Amplatzer duct occluders, and, less frequently, vascular detachable coils.^4^, ^5^, ^6^, ^7^, ^8^ The most commonly used device is the Amplatzer Duct Occluder, and also in our case, a 6/4-mm Amplatzer Duct Occluder was implanted. Compared with other devices, it offers the advantage of an invaginated tip at the disc end, likely reducing the risk of interference with the aortic valve leaflets. The unique design of the Amplatzer Duct Occluder allows the proximal part of the device to adapt to the anatomy of the fistula and assure an optimal positioning. The device was oversized by 2 to 4 mm based on both preprocedural TEE and CT scan images to further ensure long-term stability and complete closure.

Furthermore, the CT scan with 3-dimensional reconstruction helped to elucidate the anatomy of the fistulous tract, showing an elongated communication that could potentially fit an Amplatzer Duct Occluder. The CT scan also played a key role in the preprocedural planning by identifying the optimal fluoroscopic projection. In particular, in line with transaortic valve replacement practice, we were able to isolate the NCC using a cusp-overlap view, thereby optimizing the visualization of the fistula, and facilitating precise device positioning.

The use of an arteriovenous wire loop is critical to provide adequate support during advancement of the delivery system. Maintaining controlled tension on both the venous and arterial sides of the wire during this maneuver is essential. In addition, based on our experience, using the jugular venous access for device delivery enables better device alignment and more controlled delivery. The retrograde deployment was considered mandatory for the chosen closure device.

## Conclusions

Aorto-RA fistula is a rare complication of transseptal puncture. Percutaneous closure is technically feasible and straightforward. Multimodality imaging in the preprocedural work-up is essential for selecting the optimal fluoroscopic projection and choosing the best interventional approach. In line with transaortic valve replacement practice, we isolated the NCC in the “cusp overlap-like” view. A jugular venous approach with the creation of an arteriovenous wire loop facilitates device alignment and delivery.

### Ethical Statement

The case was conducted according to good clinical practice, institutional guidelines, national legal requirements, European standards, and the revised Declaration of Helsinki. The patient provided informed consent for the use of personal data.

**Take-Home Messages**

• This case highlights the importance of multimodality imaging in the planning of percutaneous aorto–right atrial closure.
• Technical skills and experience in structural practice are essential to achieve a good result.

## Funding Support and Author Disclosures

Dr Eeckhout is proctor for Abbott Vascular. Dr Lochy is a speaker/proctoring/consulting honoraria from Abbott, Occlutech, Medtronic and Boston Scientific. All other authors have reported that they have no relationships relevant to the contents of this paper to disclose.

## Appendix

Video 1 Transesophageal Echocardiography Short Axis Showing the Fistula Between the Noncoronary Cusp and the Right Atrium

Video 2 Wiring of the Fistula From the Aortic Root Using a 6-F Diagnostic Amplatzer Right 1 Catheter and a Glidewire Advantage Straight-Tip 0.035-Inch, 260-cm Wire (Terumo)

Video 3 Snaring of the Advantage Wire in the Pulmonary Artery Using an EN Snare 18 to 30 mm (Merit Medical), Advanced From the Jugular Access With a Judkins Right 4 Guiding Catheter

Video 4 Positioning of a 6/4-mm Amplatzer Duct Occluder (Abbott) Through the Venous Access
